# Supplementary material for: Perceived Importance of Types and Characteristics of Support to Informal Caregivers among Spouse Caregivers of Persons with Dementia in Sweden: A Cross-Sectional Questionnaire-Based Study
Source: Int J Environ Res Public Health. 2024 Oct 11;21(10):1348. doi: 10.3390/ijerph21101348 (PMC11506862; doi:10.3390/ijerph21101348)
Supplement: Supplementary file 1 [file ijerph-21-01348-s001.zip › ijerph-3171979-supplementary.pdf]

Table S1. Perceived importance of types of support: items, and response options

**How important is support that gives you …?**				
Information and advice about the type of help and support that is available and how to access it	Not important	Quite important	Very important	Extremely important
Information about the [dementia] disease that ELDER [partner] has	Not important	Quite important	Very important	Extremely important
Training to help me develop the skills I need to care	Not important	Quite important	Very important	Extremely important
Opportunities to enjoy activities outside of caring	Not important	Quite important	Very important	Extremely important
Opportunities to have a holiday or take a break from caring	Not important	Quite important	Very important	Extremely important
Opportunities for ELDER [next of kin] to undertake activities they [he/she] enjoy	Not important	Quite important	Very important	Extremely important
Help with planning for the future care	Not important	Quite important	Very important	Extremely important
The possibility to combine care giving with paid employment	Not important	Quite important	Very important	Extremely important
The opportunity to talk over my problems [with a professional] as a carer	Not important	Quite important	Very important	Extremely important
The opportunity to talk [online or phone] over my problems [witth a professional]as a carer	Not important	Quite important	Very important	Extremely important
Opportunities to attend a carer support group [close to home lead by a professional]	Not important	Quite important	Very important	Extremely important
Opportunities to attend a carer support group [online of phone lead by a professional]	Not important	Quite important	Very important	Extremely important
Opportunities to attend a carer support group [meetingplace for couples in the same situation]	Not important	Quite important	Very important	Extremely important
More money to help provide things I need to give good care	Not important	Quite important	Very important	Extremely important
Opportunities to spend more time with my family	Not important	Quite important	Very important	Extremely important
Help to deal with family disagreements	Not important	Quite important	Very important	Extremely important
Help to make ELDER's [next of kin] environment more suitable for caring	Not important	Quite important	Very important	Extremely important

Note: 'Not important' = 0, 'Quite important' = 1, 'Very Important' = 2, 'Extremely important' = 3.

Table S2. Perceived importance of characteristics of support: items, and response options

**How important are the following characteristics of services for you?**				
Help is available at the time you need it most	Not important	Quite important	Very important	Extremely important
The help provided fits in with your own routines	Not important	Quite important	Very important	Extremely important
Help arrives at the time it is promised	Not important	Quite important	Very important	Extremely important
Care workers have the skills and training they require	Not important	Quite important	Very important	Extremely important
Care workers treat ELDER [next of kin] with dignity and respect	Not important	Quite important	Very important	Extremely important
Care workers treat you with dignity and respect	Not important	Quite important	Very important	Extremely important
Your views and opinions are listened to	Not important	Quite important	Very important	Extremely important
The help provided improves the quality of life of ELDER [next of kin]	Not important	Quite important	Very important	Extremely important
The help provided improves your quality of life	Not important	Quite important	Very important	Extremely important
The help provided is not too expensive	Not important	Quite important	Very important	Extremely important
Help is provided by the same care worker each time	Not important	Quite important	Very important	Extremely important
Help focuses on your needs as well as those of ELDER [next of kin]	Not important	Quite important	Very important	Extremely important

Note: 'Not important' = 0, 'Quite important' = 1, 'Very Important' = 2, 'Extremely important' = 3.
